# Thinking Care Through Intersectionality: Toward a Critical Epistemology of Care in Nursing Sciences

**DOI:** 10.1111/nin.70142

**Published:** 2026-07-16

**Authors:** Aline Chenou, Valérie Wilmé, Nadia Tiberti, Erik‐André Sauleau, Sabrina Garnier‐Kepka

**Affiliations:** ^1^ Les Hôpitaux Universitaires de Strasbourg, Service des Urgences du Nouvel Hôpital Civil. Laboratoire des sciences de l'ingénieur, de l'informatique et de l'imagerie (ICube, UMR7357) Université de Strasbourg Strasbourg France; ^2^ Faculté des sciences médicales et paramédicales Université d'Aix‐Marseille Marseille France; ^3^ Les Hôpitaux Universitaires de Strasbourg, Département de Santé Publique. Laboratoire des sciences de l'ingénieur, de l'informatique et de l'imagerie (ICube, UMR7357) Université de Strasbourg Strasbourg France

**Keywords:** critical epistemology, health equity, intersectionality, nursing theory, situated knowledge, standpoint theory

## Abstract

Despite their proclaimed universality, healthcare systems produce systematic inequities shaped by intersecting axes of power, a paradox that intersectionality is uniquely positioned to address. This philosophical‑reflexive inquiry proposes repositioning intersectionality within nursing sciences not merely as an analytical framework but as the foundation of a critical epistemology of care. Drawing on feminist standpoint theory (Hartsock, Harding, and Collins) and intersectional scholarship (Crenshaw, Bilge), we identify five interrelated pillars: (1) situated knowledge as epistemic foundation, (2) interlocking power relations as analytical core, (3) reflexive positionality for strong objectivity, (4) methodological pluralism responsive to complexity, and (5) care as political praxis oriented toward social justice. Through conceptual analysis and argumentative synthesis, we demonstrate how intersectionality challenges nursing's traditional reliance on biomedical neutrality, inviting reflexive inquiry into the production of knowledge about care. This epistemological reorientation equips nursing sciences to illuminate structural inequities beyond isolated social determinants, fostering research and practice attuned to relational dynamics of power and vulnerability. Applied to French nursing scholarship, where intersectionality remains under‑theorized, this framework offers pathways for educational reform, clinical reflexivity, and methodological innovation. Ultimately, it reaffirms nursing's commitment to generating transformative knowledge that not only explains but actively contests the structural production of health inequities.

## 1 Introduction

In policy and institutional discourse, healthcare systems are often presented as universal, neutral, and guided by principles of equity. This rhetoric is reflected in global health frameworks promoted by the World Health Organization and the United Nations (Or et al. 2023; WHO and World Bank Group 2025), as well as in national healthcare traditions, including the French public hospital system. Yet a substantial body of evidence shows that health outcomes and access to care vary systematically according to race, gender, class, sexuality, age, and other social positions.

In the 1980s, American jurist Kimberlé Crenshaw introduced intersectionality to address a conceptual gap in the analysis of Black women's experiences within the American judicial system, showing how race and gender, far from operating as separate categories, compound to produce qualitatively distinct forms of oppression invisible to single‐axis framework (Crenshaw et al. 2021; Vergès 2020). Beyond its legal origins, intersectionality has since become a foundational resource across the health sciences for rethinking how structural inequities are produced and sustained (Fassa et al. 2016). Yet its adoption has remained largely methodological: its deeper implications for how nursing knowledge itself is produced and validated remain under‐theorized.

While intersectionality has gained significant traction in Anglo‐Saxon and Nordic nursing sciences during the 2020s (Sherman et al. 2023; Siira et al. 2023), its uptake remains uneven globally (notably absent from French nursing scholarship) partly due to ongoing definitional debates that persist even in contexts where the concept is institutionally recognized (Fassa et al. 2016). Beyond Anglo‐Saxon and Nordic contexts, intersectionality has also taken root in bicultural societies such as in New Zealand, where Aspinall et al. demonstrated how ethnicity, gender, culture, and national origin intersect within nursing leadership (Aspinall et al. 2021), illustrating the global reach of intersectional thinking beyond its North American origins.

Where it has been adopted, intersectionality has demonstrated considerable analytical power: it enables nursing sciences to dissect how intersecting social positions shape care experiences, refine models of vulnerability and equity, and inform intervention strategies attuned to the specificities of marginalized communities (Bauer 2014; Siira et al. 2023). Yet much of this literature treats intersectionality primarily as an analytical lens applied to discrete empirical phenomena. Less attention has been paid to its deeper epistemological implications: if knowledge is socially situated and shaped by relations of power, intersectionality cannot be reduced to a methodological supplement. It invites a fundamental reconsideration of how nursing conceptualizes objectivity, authority, and the production of knowledge about care.

Within nursing scholarship, care has long been framed as the profession's moral and epistemological core, often contrasted with cure. Contemporary debates, however, challenge this distinction, emphasizing the interdependence of caring and curing across professions. Feminist and political theories further critique early caring frameworks for naturalizing care as a feminine and universal disposition, thereby obscuring power relations and gendered divisions of labor. Concept analyses also highlight fragmented and overlapping definitions of care, shaped by organizational constraints and task‐oriented practices.

Against this backdrop, care must be understood not as a self‐evident essence but as a historically contested and structurally constrained practice, a prerequisite for any critical epistemology of care. In this article, “care” refers to nurses' capacity to engage holistically with patients across physical, psychosocial, and relational dimensions, while remaining attentive to the power relations and structural conditions that shape this engagement.

Nursing's epistemological tradition has not been inattentive to the limits of biomedical knowledge. Carper's foundational typology of four patterns of knowing (empiric, esthetic, personal knowing, and ethics) marked an early broadening of disciplinary epistemology beyond narrow empiricist accounts by legitimizing forms of knowledge irreducible to empirical verification (Carper 1978). White subsequently reviewed and extended this framework, proposing sociopolitical knowing as a fifth pattern to account for the broader political and social contexts shaping nursing practice (White 1995). Chinn and Kramer further articulated emancipatory knowing as an additional pattern explicitly oriented toward social justice, structural critique, and the transformation of unjust conditions (Chinn and Kramer 2014). Yet while these frameworks opened important space for critical and pluralist epistemologies in nursing, they do not fully theorize how intersecting axes of power (race, gender, class, sexuality, and other social positions) jointly shape the conditions under which nursing knowledge is produced, validated, and marginalized. It is precisely this gap that intersectionality, understood as an epistemological resource rather than merely an analytical tool, is positioned to address.

Before elaborating how intersectionality addresses this gap, it is necessary to specify what we mean by a critical epistemology of care. Here, a critical epistemology of care treats care not as a fixed or self‐evident concept, but as a historically situated and politically charged practice whose meanings are negotiated within relations of power. It examines how those relations, structured by intersecting axes of race, gender, class, and other social positions, determine what counts as legitimate knowledge in nursing, who produces it, and how it is mobilized in practice. Such an epistemology is explicitly oriented toward critique and transformation, seeking to expose the structural conditions of knowledge production and to foster more just, reflexive, and equity‐oriented care. This definition also responds to ongoing debates in nursing scholarship, where care and caring are increasingly recognized as contested rather than fixed concepts, requiring critical re‐examination rather than assumption as stable disciplinary foundations.

Addressing this epistemological gap requires a mode of inquiry that is itself reflexive about the conditions of knowledge production, one that neither reproduces positivist neutrality nor reduces intersectionality to a methodological checklist. Philosophical‐reflexive inquiry is particularly suited to this task: unlike empirical approaches, which presuppose a relatively stable object of investigation, it operates at the level of assumptions, categories, and power relations structuring knowledge production, a mode recognized in nursing as generative beyond empirical verification (Kim 1999). Nursing philosophy has itself long confronted positivist limits; Bender and Holmes show how the discipline's art–science dualism reflects an unexamined ontological bifurcation constraining knowledge production, pointing toward ontologically pluralist approaches (Bender and Holmes 2019). Overcoming such bifurcations requires precisely the conceptual reconfiguration that philosophical‐reflexive inquiry enables, and that repositioning intersectionality as an epistemological foundation demand. Because intersectionality is a situated and politically engaged framework, reflexivity here is not incidental but constitutive: the research's positionality is not bracketed but made analytically productive (Bilge et al. 2023) enacting the very epistemological commitments the paper theorizes.

This article argues that intersectionality should be understood as the basis of a critical epistemology of care within nursing sciences. Rather than positioning intersectionality solely as a framework for examining patient vulnerability, we propose that it reconfigures nursing's understanding of knowledge itself. Drawing on feminist standpoint theory and nursing epistemology, we identify five pillars: socially situated knowledge, interlocking systems of power, reflexive positionality, methodological pluralism, and care as political praxis oriented toward health equity.

Accordingly, this paper uses philosophical‐reflexive inquiry to examine intersectionality as an epistemology of care in nursing. Drawing on seminal works by Crenshaw, Collins, Harding, and nursing epistemologists, we conduct a conceptual analysis of core attributes, methodological tensions, and implications for disciplinary knowledge production. Through argumentative synthesis, we develop propositions for a critical epistemology of care and their relevance for nursing theory and practice.

Before further developing this argument, the next section provides our position statement before a brief historical overview of intersectionality's development and its translation into the field of healthcare, with particular focus on the French context.

### 1.1 Position Statement

The authors write from a specific social and professional location: advanced nurse practitioners, biostatistician, and medical researchers trained in emergency departments within the French healthcare system, where intersectionality remains largely absent from nursing or medical curricula and clinical discourse. This situatedness is not incidental to our argument, it is constitutive of it.

Emergency departments occupy a singular epistemic position: as the primary sites of recourse for populations whose vulnerabilities are amplified by the intersection of race, class, gender, age, migration status, and disability, they render visible the structural production of health inequities in ways that care settings oriented toward longer timeframes and intrinsically more regulated access (such as primary care or scheduled specialist consultations, which are by nature subject to referral conditions, eligibility criteria, or extended waiting periods) tend to obscure. It is from this vantage point, at the confluence of urgency, power, and vulnerability, that we have theorized intersectionality as a critical epistemology of care.

This positioning shapes what our analysis renders visible: the epistemic stakes of triage, the political dimensions of bedside assessment, by which we mean the ways in which clinical judgment at the bedside is never a purely technical act, but is traversed by implicit norms about whose pain is deemed credible, whose account is trusted, and whose urgency is recognized, norms that are themselves shaped by intersecting axes of race, gender, class, and institutional power, and the knowledge generated by proximity to structural exclusion. It may limit our grasp of contexts where intersectionality is institutionally normalized, or where care unfolds across longer therapeutic timescales. We hold this partiality as a condition of our inquiry, not a defect to be corrected, enacting the very situated objectivity we theorize.

## 2 General Historical Overview

Intersectionality emerged from a rich, transnational web of intellectual and political struggles that defies any single, linear account (Bilge et al. 2023, 111). This section traces the major lines of development that scholars conventionally recognize, foregrounding the social movements and lived experiences from which the theory drew its critical force, and acknowledging that any such account involves deliberate choices about emphasis and scope.

While the term “intersectionality” itself did not appear until much later with Crenshaw, it is worth noting that certain earlier intellectual and political work had already highlighted some of its fundamental concerns. For example, Sojourner Truth's 1851 speech “Ain't I a Woman?” (Jones et al. 1851) challenged both racist and sexist exclusions, by demonstrating that conceptual frameworks treating “woman” or “Black people” as distinct and homogeneous categories could not capture Black women's experiences. This famous speech can thus be seen as a precursor to the concerns that intersectionality would later formalize a theoretical framework.

With the introduction of the term “intersectionality,” Crenshaw and subsequent scholars working with this concept challenged earlier theoretical paradigms by recognizing that multiple axes of power and identity coexist and shape lived experience. Collins expanded this understanding with her notion of the “matrix of domination,” describing systems of oppression not as fixed hierarchies or single‐agent structures but as interlocking processes circulating within a broader social matrix where oppressions intersect (Collins 2002; Wesp et al. 2019). While Collins positioned intersectionality as a theoretical framework for elucidating the unique experiences of the multiply marginalized (Abrams et al. 2020), Crenshaw emphasized its function as an analytical tool.

Limiting the discussion to African American feminist movements alone would fail to capture its global scope. Intersectional thinking also emerged from Chicana and Latina movements (particularly in the works of Anzaldúa) as well as from Indigenous and Chinese American women, who likewise articulated the interplay of racism, sexism, and coloniality (Bilge et al. 2023, 121).

### 2.1 Intersectionality in Healthcare: A Necessary Incursion to (Re)Conceptualize Care

Public health has been at the forefront of recognizing intersectionality's potential to address health inequities, given persistent evidence that marginalized groups face both greater risks and reduced access to resources (DiPrete and Eirich 2006; Harari and Lee 2021). Since the 2010s, it has been mobilized as both a theoretical and methodological approach to analyzing relational, co‐constructed determinants beyond additive models, with calls for its integration into public health training (Bowleg 2012). Frameworks such as Hankivsky's Intersectionality‐Based Policy Analysis (IBPA) further operationalize this perspective by examining how policies produce differential inclusion and exclusion (Hankivsky et al. 2014).

From the 2010s onward, pioneering authors in nursing sciences began to appropriate this concept, using it as a powerful tool to transform nursing practice into a more just and inclusive discipline, one that acknowledges the dynamics of power and privilege embedded at the very heart of care and seeks to promote social justice (Van Herk et al. 2011). Van Herk et al. stand among one of the first scholars to examine intersectionality within the nursing profession; a decade later, Aspinall et al. were the first to apply this framework specifically to nursing leadership (Aspinall et al. 2021), while the 2023 integrative review further consolidated the history of intersectionality in nursing literature (Aspinall et al. 2023).

Intersectionality resonates so profoundly in nursing sciences because it explains and illuminates, at all levels, the relationships between systems and how these impact individuals (Van Herk et al. 2011), including their health and well‐being. Thus, for example, in preliminary works in nursing sciences adopting this concept, the intersectional lens is employed to explain how the stigmatization of people living with HIV/AIDS is often interwoven with other experiences of marginalization, such as gender, socioeconomic status, culture, and sexuality (Mill et al. 2009; Van Herk et al. 2011).

While these early applications positioned intersectionality primarily as an analytical or methodological tool, its potential to reconfigure the epistemological foundations of nursing knowledge remains largely unexplored.

### 2.2 French Situation

Within nursing science, the concept of intersectionality began to gain considerable prominence during the 2020s, especially across Anglo‐Saxon and Nordic countries (Aspinall et al. 2023; Sherman et al. 2023; Siira et al. 2023). The relative marginalization of intersectionality within French nursing scholarship offers a revealing case of how epistemic hierarchies shape disciplinary development.

In the French academic world, the emergence of this concept remains almost absent from the nursing literature. This absence reveals a major gap, given that intersectionality is likely to become increasingly prominent in the coming years as a conceptual tool for rethinking care practices.

This limited adoption may partly be explained by the ideological divide surrounding the reception of intersectionality in France, caught between the universalist republican discourse and the depoliticization of nursing science. Indeed, the very term intersectionality has, in recent years, sparked intense public controversy in France, reaching the political sphere and being associated with so‑called “woke” ideologies (Lépinard and Mazouz 2021). This media‐driven appropriation has contributed to the disqualification of the concept in certain academic circles, despite its transformative potential for envisioning a more equitable healthcare system.

Even within academic contexts where intersectionality is acknowledged and integrated, debates continue regarding how the concept should be properly defined (Fassa et al. 2016).

The relative marginalization of intersectionality within French nursing scholarship can be read not as a simple temporal delay, but as a revealing illustration of broader epistemic hierarchies structuring health sciences. Within a context historically shaped by biomedical dominance, legitimate knowledge has largely been defined through positivist criteria privileging neutrality, standardization, and universality. Critical theories, particularly those explicitly grounded in social justice and power analysis, often encounter resistance, being perceived as ideological rather than scientific. This configuration maintains disciplinary boundaries that both marginalize nursing knowledge and pressure it to conform to biomedical norms of legitimacy.

From this perspective, the limited uptake of intersectionality in French nursing sciences exemplifies how hierarchies of knowledge regulate which frameworks are deemed admissible within academic spaces. Intersectionality unsettles these hierarchies precisely because it exposes the political conditions of knowledge production and challenges the fiction of epistemic neutrality. Its reception, or resistance, thus becomes symptomatic of deeper tensions between technocratic models of care and critical epistemologies oriented toward social justice. Situating this analysis within the French context does not provincialize the argument; rather, it renders visible how local configurations of power illuminate global dynamics shaping the development of nursing scholarship.

Yet this resistance is not merely political. It is also epistemological, and partly sustained by the concept's own definitional instability. Before intersectionality can be mobilized as an epistemological foundation, the ambiguities that render it susceptible to appropriation, dismissal, or depoliticization must be squarely confronted.

## 3 Deconstructing Misconceptions About Intersectionality: Misunderstandings, Clarifications, and Definitions

Intersectionality as a theory disrupts established codes and often crystallizes tensions and academic debates. Indeed, even qualifying it as a “theory” remains uncertain, with some authors preferring terms such as “analytical framework,” “intersectional lens,” “conceptual framework,” or “concept” over “theory” (Siira et al. 2023) because each framing implies a different understanding of knowledge, method, and politics. This question of how to name intersectionality is far from merely semantic. Each designation carries distinct epistemological commitments that shape how intersectionality can be mobilized within a discipline (Cho et al. 2013; Siira et al. 2023). Framing intersectionality as a theory, in its positivist sense, implies a systematized body of propositions subject to empirical testing and potential falsification, a framing that risks assimilating it into the positivist epistemological tradition it was, in part, developed to contest (Bilge et al. 2023). Positioning it as a lens, while widespread in applied health literature, risks reducing it to a descriptive optic that can be selectively adopted or set aside without deeper epistemological commitment, thereby evacuating critical and counter‐hegemonic dimensions that early intersectional thinkers regarded as non‐negotiable, when mobilized without its underlying epistemological commitments (Cho et al. 2013; Collins 2002; Crenshaw et al. 2021). The designation of analytical framework, by contrast, better reflects intersectionality's epistemological roots in Black feminist thought: it foregrounds its function as a situated, reflexive, and politically engaged tool for interpreting how axes of power operate simultaneously and relationally, producing effects irreducible to any single axis. This distinction carries direct implications for the argument advanced in this paper. If intersectionality were merely a theory, its contribution to nursing science would be additive, one more resource to be incorporated without reconfiguring the discipline's epistemological foundations. If it were a lens, it could be instrumentalized without altering how nursing conceptualizes objectivity, authority, or the conditions of valid knowledge. But, understood as an analytical framework grounded in feminist standpoint epistemology, intersectionality does something more fundamental: it reorients the very conditions under which nursing knowledge is produced, validated and mobilized, which is precisely what a critical epistemology of care demands (Harding 1991; Reed 2022).

Intersectionality's academic institutionalization has not been without contestation. Several scholars argue that its incorporation into mainstream academic and policy discourse has blunted its counter‐hegemonic edge, producing a depoliticized version that stands in direct opposition to what early thinkers advocated, and that risks perpetuating the very inequalities it was developed to expose (Bilge et al. 2023; Richman and Zucker 2019). Despite these tensions transversal elements have emerged across disciplines that are directly consequential for this paper's argument. Intersectionality posits that individuals occupy social positions shaped by the simultaneous operation of multiple axes of power, embedded in historically situated relations of domination and privilege (Abrams et al. 2020). Six recurring themes underpin this framework: social inequalities, interlocking power relations, social context, relationality, social justice, and complexity (Bilge et al. 2023; Siira et al. 2023). These themes are not merely descriptive categories, actually, they constitute the epistemological scaffolding through which intersectionality challenges how knowledge is produced and for whom. Foregrounding relationality and social context, intersectionality insists that knowledge cannot be abstracted from the conditions of its production, centering complexity and social justice, it orients inquiry toward transformation rather than neutral description. It is precisely this architecture, not any single theme in isolation, but their interlocking logic, that positions intersectionality as a critical epistemology of care rather than a simple analytical tool.

Grounded in the epistemological foundations, intersectionality can now be examined as an operation framework, one that not only informs how knowledge is produced in care settings but also guides how research and practice can be structured to actively advance health equity. Its ongoing tension between critical stance and institutional assimilation is not a weakness but a site of heuristic potential, the very place where intersectionality's capacity to interrogate the conditions of knowledge production remains alive and consequential.

## 4 Intersectionality as a Critical Epistemology of Care

Many intersectionality theorists share epistemological affinities with feminist standpoint theory (Bilge et al. 2023; Lépinard and Mazouz 2021). In feminist theories, standpoint theory emerged with Nancy Hartsock's publication “The Feminist Standpoint” developed within a Marxist lineage. Hartsock proposed that oppressed groups occupy a privileged vantage point for understanding mechanisms of domination. So, their experiences provide a critical and comprehensive vision of the social order. In this article, Hartsock shifts this perspective by emphasizing that women's standpoint, shaped by gender social relations, constitutes a specific source of knowledge about the world (Flores Espínola 2012).

Building on this foundation, Sandra Harding deepened reflection on the socially situated character of knowledge. For Harding, all knowledge arises from a specific social context, necessitating identification of social positions yielding the most favorable conditions for expanded objectivity. In Whose Science? Whose Knowledge? (1991), she advanced the notion of “strong objectivity,” which demands explicit recognition of the knower's social position. According to Harding and feminist standpoint scholars, the objectivity of research is strengthened when analysis departs from women's experiences but is further bolstered when positioned from the standpoint of historically oppressed or marginalized women (Flores Espínola 2012; Fonte and Lelaurain 2024; Harding 1991). Extending Harding's work, Patricia Hill Collins broadens the scope of standpoint epistemologies by incorporating an intersectional dimension. In Black Feminist Thought, Collins demonstrates that African American women, positioned at the crossroads of racism and sexism among other oppressions, develop a singular form of knowledge arising from their collective experiences of oppression and resistance, enabling a situated communal understanding (Coenga‐Oliveira 2018; Collins 2002).

Nurses are intimately familiar with this dialectic between power and subordination. They occupy a particular position within healthcare (one sometimes described as “oppressed”) which grants access to forms of experiential knowledge otherwise invisible in dominant biomedical hierarchies (Siira et al. 2023). Far from equating nurses with historically oppressed groups, their professional location in healthcare affords them a situated understanding of power dynamics and structural inequities. From this vantage, nurses can act as advocates for health equity, challenging injustices through praxis that ranges from bedside care to policy engagement (Siira et al. 2023). These theoretical insights illuminate nurses' epistemic positioning within healthcare, where their practice is shaped by the intersection of institutional power and lived care relations. Consider, for instance, an ED nurse assessing cumulatively vulnerable patients whose unplanned visits reveal intersectional compounding beyond isolated risk factors. Venkatesh et al. demonstrate how multiple chronic conditions, dual Medicare–Medicaid eligibility (proxy for extreme socioeconomic precarity), and skilled nursing facility residence interact to yield exponentially elevated ED visit rates, incidence rate ratios rising from 12.23 to 23.8 expected visits/year. These non‐additive effects affirm nursing's epistemic access to structural amplification patterns invisible to unidimensional models, positioning frontline assessment as a critical site for intersectionality‐informed care (Venkatesh et al. 2020).

This proximity, often mischaracterized as marginal within healthcare hierarchies, grants nurses a distinctive understanding of processes of vulnerabilization and unequal access to care. Forged in practice and relationship, their knowledge transcends technical expertise to become an epistemic resource for revealing structural exclusion and informing equitable intervention. At the frontline of care, nurses' vantage point constitutes a privileged observatory of power relations, generating socially grounded, user‐centered knowledge. This socially situated epistemology exemplifies how intersectionality can underpin a critical epistemology of care, one that transforms lived experience into a source of reflexive, justice‐oriented knowledge.

### 4.1 Toward a Critical Epistemology of Care

For many intersectional scholars, including Bilge and Collins, intersectionality functions as a critical praxis that locates frontline professions such as teaching and nursing at the center of social intervention. Bilge and Collins explicitly identify nurses as professionals whose experiences of violence, homelessness, hunger, illiteracy, poverty, and sexual violence may generate distinctive insights into how inequalities shape social problems and unequal exposure to harm (Bilge et al. 2023, 83). They also stress that, for these professions, intersectionality is not merely a heuristic lens but an essential intervention strategy. Extending this argument, we contend that in nursing sciences, intersectionality has implications beyond practice alone: it offers a way to rethink the epistemic foundations of care, including how knowledge is produced, validated and mobilized in clinical and research setting.

This view lays the groundwork for conceiving nurses' epistemic position as central to a critical epistemology of care. By this, we mean an approach that treats care not as a neutral of self‐evident nursing essence, but as socially situated, politically shaped, and epistemically contested practice whose meanings and legitimations are produced with relations of power. In nursing, this matters because what counts as legitimate care knowledge is never independent from professional hierarchies, institutional norms, and broader structures of inclusion and exclusion. While care is often portrayed as universal, the nursing literature has increasingly shown that it is also a contested concept, one whose epistemic status cannot be taken for granted. An intersectional perspective helps make these tensions visible by showing how gender, race, class, sexuality, age, ability and other social positions shape both care practice and care experience. De Sousa and Varcoe further reinforce this critical reorientation by centering Black feminist thought in nursing praxis and arguing for a transformation of nursing practice that recognizes intersectionality and affirms the knowledge of historically excluded groups (De Sousa and Varcoe 2022).

From this standpoint, a critical epistemology of care becomes imperative. It articulates a reflexive standpoint posture with an intersectional methodology to examine how these social positions shape both the production of nursing knowledge and its application in practice. Aspinall et al. (2021) illustrate this in showing that leadership and workplace inequalities in healthcare cannot be understood without accounting for the interaction of ethnicity, gender, culture and national origin (Aspinall et al. 2021). Likewise, Agénor et al. demonstrate that disparities in cervical‐cancer screening are not reducible to a single axis of difference but emerge from the intersecting experiences of sexual orientation, social position and healthcare access (Agénor et al. 2014). Crucially, these studies do more than document inequities, they also show that what counts as visible, relevant, or actionable knowledge in nursing depends on whether intersecting social positions are analytically taken into account. In this sense, they illustrate how intersectionality reshapes the production of nursing knowledge by revealing phenomena that remain obscured within single‐axis or additive approaches.

Seen in this light, a critical epistemology of care matters for nursing because it repositions care as a site of inquiry, critique, and transformation rather than as a fixed disciplinary giver. It also allows nursing science to connect epistemic questions to social justice, by showing how knowledge about care is produced, validated, and mobilized in ways that can either reproduce or challenge inequity.

### 4.2 Quantitative Methodology: Bayesian Inference as a Promising Alignment

The methodological tension surrounding intersectionality often crystallizes in questions of research design: how can methods honor its ontological complexity, retain its critical orientation, and still meet scientific standards? This tension is not merely technical, it is fundamentally epistemological. Conventional quantitative frameworks, built on universalizing logics of abstraction and value‐neutrality, sit uneasily with an epistemology that foregrounds situatedness, power, and the relational constitution of social position. A persistent pitfall lies in quantitative studies that “add up identities” through simplistic interaction terms, thereby missing how intersecting positions create emergent, context‑specific effects (Abrams et al. 2020). This section argues that Bayesian inference does not simply accommodate intersectionality methodologically, it enacts its core epistemological commitments.

This enactment becomes legible against the backdrop of existing quantitative limitations. Reviews show that many studies invoking intersectionality limit their analyses to two or three social dimensions, with weak theoretical grounding and limited policy relevance (Bauer et al. 2021). This reflects a structural tension: frequentist frameworks, built around binary hypothesis testing and additive interaction terms, privilege abstraction over contextual embeddedness. Their implicit claim to value‐neutrality, what Harding calls “weak objectivity,” a posture that scrutinizes research objects while leaving the normative assumptions of the knowing subject unexamined and unaccountable.

To understand why Bayesian inference constitutes a more epistemologically coherent option, it is necessary to return to the situated knowledge thesis at the heat of feminist standpoint epistemology (Haraway 1988; Harding 1991). This thesis holds that knowledge is always produced from a particular social location, and that acknowledging this positionality, rather than suppressing it, yields stronger, less distorted accounts of social reality. Crucially, Bayesian inference operationalizes precisely this logic: the researcher must explicitly specify prior distribution, encoding positional assumptions about the phenomena before encountering data. These priors are not noise to be minimized but constitutive elements of the knowledge‐production process, made visible and open to critical scrutiny. In this sense, the Bayesian updating process, moving from prior to posterior through iterative encounter with evidence, formally mirrors what standpoint theorists describe as the reflexive production of situated knowledge.

This epistemological alignment is not merely metaphorical. Where frequentist inference claims to bracket the researcher's standpoint Bayesian inference requires its articulation. This reflexive transparency resonates with Harding's concept of “strong objectivity,” which holds that scientific rigor is strengthened, not compromised, by making the researcher's positional assumptions explicit and subject to critique. Rather than feigning a view from nowhere, Bayesian models enact what Haraway called “situated knowledge”: partial embodied and accountable. The prior is not a confession of bias, it is a theoretically grounded assertion about the world, revisable through evidence and transparent to the interpretive community. Bayesian inference therefore represents a promising, though not exclusive, methodological alignment with a critical epistemology of care (Espinosa et al. 2025). By revealing the normative assumptions embedded in analytical choices and by enabling models sensitive to heterogeneity and complexity, Bayesian methods exemplify how quantitative research can enact methodological pluralism attuned to power, context, and reflexivity. Yet they remain one among several quantitative strategies compatible with intersectional inquiry (Mahendran et al. 2022) reaffirming that methodological choice is itself a situated act of knowledge production.

The methodological pluralism made possible by Bayesian approaches illustrates that epistemological commitments are always enacted through concrete research choices. Moving from these methodological considerations, the next section consolidates the theoretical foundations of a critical epistemology of care and articulates its five guiding pillars.

## 5 Propositions for a Critical Epistemology of Care

A critical epistemology of care is proposed here as a disciplinary metatheory for nursing sciences, a systematic account of how nursing knowledge is produced, validated, and put to work, one that takes the intersecting structures of power shaping health as both its explanatory object and its normative horizon. Rather than competing with mid‐range nursing theories (e.g., Orem, Watson), it interrogates the epistemological conditions under which knowledge claims in nursing may be rendered visible, accountable, and equity‐oriented. It is neither a research method nor a clinical protocol, but the philosophical ground from which both derive their coherence.

Building on this discussion, intersectionality can be understood not simply as an analytical framework but as the foundation of a critical epistemology of care, reorienting how nursing knowledge is produced, validated, and mobilized. This epistemology is structured around five interrelated pillars: situated knowledge, interlocking power relations, reflexive positionality, methodological pluralism, and care as political praxis.

Table 1 outlines these pillars and their implications for nursing scholarship. Although illustrated through emergency nursing, a context of heightened intersectional visibility due to its role as a primary access point for marginalized populations, this framework is not context‐specific but applicable across all domains of nursing where care is shaped by intersecting power relations.

**Table 1:** The five pillars of a critical epistemology of care, synthesizing feminist standpoint theory, and intersectionality for nursing sciences.

Pillar	Foundational principle	Implications for nursing sciences	Clinical illustration
1. Situated knowledge	Rejects neutrality; knowledge emerges from specific social positions (Harding, Collins)	Clinical and relational experience constitutes a primary, not supplementary, epistemic source, legitimizing knowledge generated at the bedside as theoretically productive, not merely illustrative	An ED nurse's repeated encounters with homeless patients generate epistemic access to masked chronic pain invisible to standardized triage instruments, enabling timely clinical escalation
2. Interlocking power relations	Axes of oppression intersect within historically situated matrices of domination (Crenshaw, Bilge)	Health disparities must be modeled as products of co‐constitutive structural forces, not aggregated individual attributes, shifting the analytical unit from identity to power configuration	A racialized, low‐income woman's chest pain is systematically under‐triaged through the compounded effect of gender and race (Coisy et al. 2024)
3. Reflexive positionality	Requires explicit acknowledgment of the knower's social and institutional location as a condition of strong objectivity (Harding)	Requires nurses and researchers to make their institutional, professional, and biographical positioning analytically explicit, transforming reflexivity from a methodological courtesy into an epistemological obligation	A nurse interrogates her own moral framing during a pain assessment, recognizing that naming her positionality, rather than suppressing it, strengthens, rather than undermines, her clinical objectivity
4. Methodological pluralism	Methods must be coherent with intersectional ontology, capable of modeling relational complexity	Mandates methodological choices governed by ontological coherence rather than disciplinary convention, prioritizing designs that model interaction effects and non‐linear co‐constitution across multiple social positions simultaneously	Hierarchical Bayesian models stratified by race × gender × age × insurance status reveal pain management inequities structurally invisible to either ethnographic or additive regression approaches alone
5. Care as political praxis	Knowledge production inseparable from transformative action toward health equity	Nursing knowledge is normatively bound to transformative action: research findings, clinical observations, and advocacy are continuous rather than sequential activities	A nurse systematically documents disproportionate “Left Without Being Seen” rates among elderly migrants, conducts an intersectional analysis, and successfully advocates for a bilingual community health worker at triage

### 5.1 Situated Knowledge as Epistemic Foundation

A critical epistemology of care begins by identifying where legitimate nursing knowledge originates. Drawing on feminist standpoint theory, it rejects both the notion that objectivity requires erasing the knower's position and the broader epistemological fiction of the view from nowhere. On the contrary, beginning from the lived experiences of those who inhabit marginalized or subordinated locations, patients and communities, but also nurses positioned within biomedical hierarchies, yields more comprehensive and less distorted understandings of health and care. Knowledge produced from these socially and institutionally situated positions, particularly by nurses embedded in sustained relational encounters with patients at the margins of care, generates epistemic access that standardized instruments systematically foreclose. For nursing sciences, this pillar authorizes clinical experience, relational attunement, and community‐embedded insight as primary theoretical sources, not merely as illustrations of concepts derived elsewhere. The foundational question it addresses is therefore: where does nursing knowledge come from, and whose experience counts as evidence?

### 5.2 Interlocking Power Relations as Analytical Core

Intersectionality underscores that systems of power do not operate independently. Gender, race, class, sexuality, age, ability, and other social positions intersect within historically and geographically specific matrices of domination. A critical epistemology of care therefore shifts the analytical lens from individual categories to interlocking power relations. Health disparities cannot be explained through additive identity models; they must be examined as products of dynamic structural forces shaping clinical encounters, professional hierarchies, and policy frameworks. For nursing sciences, this entails interrogating not only patient experiences but also the epistemic hierarchies and organizational structures that define care itself.

### 5.3 Reflexive Positionality and Strong Objectivity

Acknowledging that knowledge is situated raises an immediate epistemological obligation: if the knower's position shapes what is knowable, then that position must be made visible and analytically accountable. This is the specific contribution of reflexive positionality, it regulates the conditions under which nursing knowledge is validly produced. Following Harding's concept of strong objectivity, scientific rigor is not achieved by suppressing the researcher's or clinician's location but by subjecting it to critical scrutiny. In nursing research, this requires examining how professional status, disciplinary training, and institutional affiliation shape research questions, interpretive choices, and clinical judgments. Crucially, this pillar is not reducible to the first: situated knowledge establishes that position grants epistemic access, and reflexive positionality establishes that unexamined position introduces systematic distortion. Together, they are complementary but logically distinct.

### 5.4 Methodological Pluralism Responsive to Complexity

The ontological commitments established by the first three pillars carry direct methodological implications, and it is this translation from philosophical ground to empirical practice that the fourth pillar addresses. Conventional quantitative approaches rooted in additive regression models cannot capture the non‐linear, co‐constitutive dynamics that intersectionality posits; qualitative approaches, while offering unmatched access to lived experience, require explicit theoretical scaffolding to move from situated account to structural analysis. A critical epistemology of care therefore endorses methodological pluralism, not as eclecticism, but as principled alignment between ontological commitments and research design. Mixed‐methods architectures, causal mediation models attentive to mediating mechanisms, and probabilistic strategies such as hierarchical Bayesian inference capable of modeling interaction effects across multiple social positions are preferred not for their technical sophistication but because they honor the relational logic that this epistemology demands. The goal is not to elevate one methodological tradition over another but to ensure that research designs treat intersecting social positions as embedded in power structures rather than as isolated variables. The criterion for methodological choice is coherence with intersectional ontology, not disciplinary convention.

### 5.5 Care as Political Praxis Oriented Toward Social Justice

Where the four preceding pillars establish the conditions of nursing knowledge (its sources, its object, its validity, and its methods), the fifth pillar addresses its orientation: what nursing knowledge is ultimately for. Intersectionality emerged from political struggles against interlocking oppressions, integrating it into nursing sciences without preserving its transformative orientation reduces it to a descriptive taxonomy and evacuates its critical force. Care is not a neutral technical act but an activity constituted by asymmetric relations and institutional power, and acknowledging this transforms nursing research and practice into sites of political engagement oriented toward health equity. A critical epistemology of care therefore affirms that research findings, clinical observations, and institutional advocacy are not sequential activities but continuous dimensions of a single epistemic practice. This does not entail moralization or partisan alignment. It affirms, more precisely, that in a field constituted by structured vulnerability, the claim to produce knowledge without orientation toward justice, recognition, and redistribution is itself a political position, one that a critical epistemology of care refuses. Advocacy, empowerment, and structural critique are thus not peripheral virtues of nursing but constitutive dimensions of its epistemological horizon. A critical epistemology of care ultimately reframes nursing science as a discipline committed not only to understanding vulnerability but also to transforming the structural conditions that sustain it.

## 6 Transforming Nursing Sciences: Lines of Inquiry

Having articulated the foundational pillars of a critical epistemology of care, the question now becomes how such an orientation can concretely transform nursing as a discipline and a practice. The following lines of inquiry outline avenues through which intersectionality may inform education, clinical work, and research, advancing nursing science toward greater reflexivity and social justice.

### 6.1 Education

In both initial and continuing education, curricula should include intersectionality training grounded in critical inquiry rather than prescriptive moral injunction, what we term an approach free from moralizing discourse. By this, we mean a pedagogy that examines, for example, how a nurse's unreflective use of the term “frequent user” to describe a homeless patient with recurrent emergency presentations encodes both class bias and epistemic dismissal, without reducing the educator's role to assigning blame. The goal is structural literacy, not individual condemnation. Such education fosters critical reflection on power and privilege from the outset of professional preparation, enabling students and instructors alike to understand their own positionality within broader social structures (Siira et al. 2023). Reflexive positionality, as a condition of strong objectivity, must become a pedagogical practice, not an optional module, one that requires educators themselves to engage in explicit examination of how their own social location shapes what they teach as legitimate nursing knowledge. Concretely, this might take the form of structured reflexivity exercises embedded in simulation debriefs, where students are asked to articulate how their own social location may have shaped their clinical reasoning, for instance, whether assumptions about a patient's pain tolerance were influenced by their perceived ethnicity or socioeconomic background. This reflective awareness may ultimately reshape how nurses perceive and enact their professional roles, both within institutions and at the bedside.

### 6.2 Clinical Practice

Identifying inequities in care delivery remains a pressing challenge for patients and healthcare systems alike. Empirical evidence confirms that care‑related discrimination persists, including in France. For instance, Coisy et al. (2024) recently demonstrated how cognitive biases in clinical reasoning, linked to patients' skin color and gender, affect triage decisions for chest‑pain cases in emergency. Although based on simulated scenarios, their findings are revealing. Similarly, age‑based discrimination, or ageism, remains one of the most normalized forms of bias in health contexts, with many patients reporting self‑perceived experiences of exclusion (Dobrowolska et al. 2019). In an aging world, recognizing and addressing ageism will likely become a central imperative for future health systems.

These findings are not merely empirical observations to be corrected by better protocols; they are symptomatic of the epistemic hierarchies that a critical epistemology of care seeks to dismantle, hierarchies that render certain bodies, particularly at the intersection of race, gender, and class, as less credible sources of knowledge about their own pain. Practically, this might be operationalized through structured professional practice analysis, where clinical teams collectively revisit triage decisions involving patients whose compounding identity markers (age, gender, ethnicity, and socioeconomic status) may have shaped care allocation. Unlike protocol‐based audit tools, this reflective approach situates intersectional awareness within a relational, deliberative process, more congruent with the epistemological commitments outlined in this paper.

### 6.3 Research

Nursing research can re‑formulate its questions through an intersectional lens to uncover areas of invisibility and discrimination in care delivery. Such reframing bears directly on the pursuit of health equity, encouraging studies that examine how multiple social determinants converge to shape experiences of vulnerability and resilience. Methodologically, this reorientation demands designs that honor intersectionality's relational ontology. As argued, hierarchical Bayesian models offer one such avenue, enabling simultaneous analysis of intersecting positions without reducing them to additive variables. For example, rather than studying “elderly patients” as a monolithic group, an intersectional study design might examine how older women of immigrant background experience pain recognition differently than older men of majority background within the same emergency department. This methodological choice is itself an epistemological act.

### 6.4 Advocacy and Leadership

In increasingly complex contexts, nursing advocacy and leadership are essential, as political decisions continuously shape clinical practice. Sustaining a virtuous cycle between research and practice ensures that nursing contributes to policy while amplifying patients' voices without substituting them, in an ethics of solidarity. Nowhere is this more evident than in emergency settings, where nurses, positioned at the frontline of structural exclusion, act not only as witnesses but as credible narrators and challengers of injustice.

Understood as a critical epistemology of care, intersectionality does more than extend nursing's analytical tools: it redefines its relationship to knowledge, justice, and action, grounding a commitment to contesting health inequities. In this perspective, advanced practice nurses, at the interface of clinical and organizational decision‐making, are uniquely positioned to operationalize these insights, for example, by advocating for the integration of intersectionality indicators into hospital quality dashboards.

## 7 Conclusion

Intersectionality unsettles disciplinary boundaries by foregrounding marginalized perspectives and challenging hierarchies of expertise, positioning itself as both an analytic framework and a normative commitment to equity and justice. For nursing science, this implies sustaining a critical dialogue between praxis and inquiry, where intersectionality functions not as a conceptual add‐on but as a means of rendering visible the blind spots of care, institutions, and professional hierarchies.

Situated at the frontline of care yet embedded in biomedical power structures, nurses occupy an ambivalent position that confers not moral authority but epistemic responsibility. This vantage point enables critical interrogation of how inequalities are produced and enacted in everyday care. Understood as an epistemological framework, intersectionality reframes vulnerability as the situated product of intersecting structural forces, accessible only through reflexive and situated inquiry.

The challenge, then, is not merely to invoke intersectionality but to operationalize it across research design, methods, analysis, and knowledge co‐production, while interrogating researchers' own positionalities. In this sense, integrating intersectionality does more than refine nursing knowledge: it reorients the discipline toward a critically engaged, socially accountable epistemology of care.

## Funding

The authors have nothing to report.

## Ethics Statement

The authors have nothing to report.

## Conflicts of Interest

The authors declare no conflicts of interest.

## Disclosing the Use of Artificial Intelligence (AI)

The authors used an AI‐assisted tool (Perplexity AI) for the purpose of translating selected passages from French into English. All translated content was subsequently reviewed, verified, and approved by the authors. The authors take full responsibility for the accuracy and integrity of the published work. AI was not used for the generation of ideas, arguments, or conclusions presented in this manuscript.

## Data Availability

Data sharing not applicable to this article as no datasets were generated or analyzed during the current study.
